# Slower Visuomotor Corrections with Unchanged Latency are Consistent with Optimal Adaptation to Increased Endogenous Noise in the Elderly

**DOI:** 10.1371/journal.pcbi.1000708

**Published:** 2010-03-12

**Authors:** Michael Sherback, Francisco J. Valero-Cuevas, Raffaello D'Andrea

**Affiliations:** 1 Institute for Dynamic Systems and Control, ETH-Zurich, Zurich, Switzerland; 2 Department of Biokinesiology and Physical Therapy, University of Southern California, Los Angeles, California, United States of America; 3 Department of Biomedical Engineering, University of Southern California, Los Angeles, California, United States of America; Northwestern University, United States of America

## Abstract

We analyzed age-related changes in motor response in a visuomotor compensatory tracking task. Subjects used a manipulandum to attempt to keep a displayed cursor at the center of a screen despite random perturbations to its location. Cross-correlation analysis of the perturbation and the subject response showed no age-related increase in latency until the onset of response to the perturbation, but substantial slowing of the response itself. Results are consistent with age-related deterioration in the ratio of signal to noise in visuomotor response. The task is such that it is tractable to use Bayesian and quadratic optimality assumptions to construct a model for behavior. This model assumes that behavior resembles an optimal controller subject to noise, and parametrizes response in terms of latency, willingness to expend effort, noise intensity, and noise bandwidth. The model is consistent with the data for all young (n = 12, age 20–30) and most elderly (n = 12, age 65–92) subjects. The model reproduces the latency result from the cross-correlation method. When presented with increased noise, the computational model reproduces the experimentally observed age-related slowing and the observed lack of increased latency. The model provides a precise way to quantitatively formulate the long-standing hypothesis that age-related slowing is an adaptation to increased noise.

## Author Summary

In a hand-eye coordination task that requires continuous movement to correct for a disturbance, it turns out that signs of response to the disturbance appear no later in the elderly than in the young. The elderly motion is noisy and less efficient, however, and once movements in response to a disturbance begin, they are at a lower speed. One can model subject response by assuming that it results from combining noise and a response that is mathematically optimal given this noise, delay, and a least-squares sort of control objective. This modeling approach is appropriate for young and most elderly subjects. The model holds that increased noise should lead to no change in delay until response gets underway, but should make the response itself proceed at a slower speed. This is consistent with the data and with a causal link from the observed noise and disorder in elderly motor function to the observed age-related slowing.

## Introduction

The existence of a general phenomenon of age-related impairment of the sensorimotor system is widely accepted [1]–[3], but its causes are incompletely understood and its expression varies depending on task. Conventional wisdom is that impairment is the result of slowing and increased sensorimotor noise. In this paper we show that in a compensatory tracking task, age-related slowing is confined to the later phases of the response, and that this slowing is consistent with the adaptation of a relevant optimal control strategy to increased noise. This suggests that slowing in this task is consistent with adaptation to the cause of impairment, i.e. increased noise, rather than a cause of impairment.

The hypothesis that age-related slowing is an adaptive response to increased cortical disorder rather than a primary cause of impairment was put forward in a survey of work on aging and reaction times [2]. It is suggested in [2] that the elderly average sensory data using longer timescales in order to reduce the effect of cortical noise. Recent data confirms that age related slowing is correlated with increased disorder in cortical Event Related Potentials [4],[5], and in [5] the same hypothesis about averaging noise away is formed. In [6] data is presented suggesting that slowing is too great to be accounted for by then-current empirical speed-accuracy tradeoff models. In [7] the modeling concepts of submovements and signal-dependent motor variability [8] were used to analyze age effects in a target acquisition task and the authors suggested that elevated motor noise is a primary cause of slower performance, based on empirically parametrized speed-accuracy tradeoffs. In this work we use a continuous compensatory tracking task and a model based on Bayesian and quadratic optimality consistent with past work on the sensorimotor application of this theory [9]–[26]. This modeling approach provides a quantitative way to relate noise levels to the timescales involved in averaging data. Our task elicits responses in the form of continuous time series that are dynamically rich compared to, for example, static force production or reaction time data. It also resembles dynamic activities of daily living such as driving a car on a windy day. The nature of the controlled dynamics and perturbations enable use of well-developed optimal control theory.

Age-related sensorimotor impairment and slowing have been heavily investigated [1]–[3],[27]. One hypothesis is that of generalized slowing, as reviewed in [1],[28]. Published results show wide variability. For example, some experiments involving simple reaction time [4],[5] fail to show a significant age-related increase, but slowing is strongly expressed in choice reaction experiments [4],[5] and is the rule rather than the exception experimentally [2]. Subtle changes in force stimuli can evoke or eliminate age effects on response latency during fingertip force generation [29]. Age-related degradation and delay in the peripheral components of the sensorimotor system has been documented in cutaneous mechanoreceptors [30], transmission of signals from visual to motor areas [31], and basic visuomotor processes such as saccades [32]–[34]. Nevertheless, it has been known for decades that age-related sensorimotor impairment in common experimental paradigms is dominated by central and not peripheral effects [2],[27].

**Figure 1:**
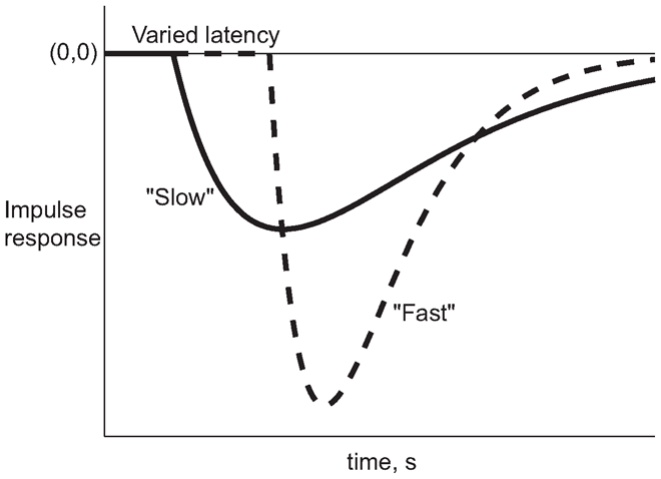
Slowing and latency. This figure gives an example of the distinction between slowing of the post-onset response vs. response latency. We use this distinction to analyze a more complex visuomotor response. The figure shows the response of two different systems to an impulse at time 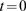. Both systems consist of delays in series with second-order viscoelastic systems. The solid line shows a shorter delay in the onset of response (less response latency) but longer settling time (slower post-onset response). The dashed line shows the converse.

We distinguish between two different kinds of “slowing” that are well characterized in the engineering field: (i) response latency, that is, delay until the onset of motor response, vs. (ii) slowed timescales of post-onset response. These are shown in Fig. 1. This distinction is closely related to the classical behavioral classification of response in reaction time studies into “reaction time” vs. “movement time” after the onset of movement [35], but we avoid this terminology to avert confusion with other published uses of those terms. Our analysis is specifically designed to disambiguate between these two kinds of slowing using complementary analytical approaches. The first is a cross-correlation approach that is strictly data-driven, phenomenological and free of assumptions; and the second is a model-based computational approach based on justifiable assumptions of optimal control theory. The advantage of the model-based approach is that it improves precision and provides a framework for mechanistically relevant analysis of response.

It is important to note that our definition of endogenous noise is a more general concept than physiological motor noise. For the purposes of this paper we define endogenous noise to be any deviation from optimal behavior (possibly of sensory, motor, conduction, or neural processing origin). We address the roles of specific sources such as muscular “motor noise” or cortical “functional dysregulation” [4] in the Discussion.

**Figure 2:**
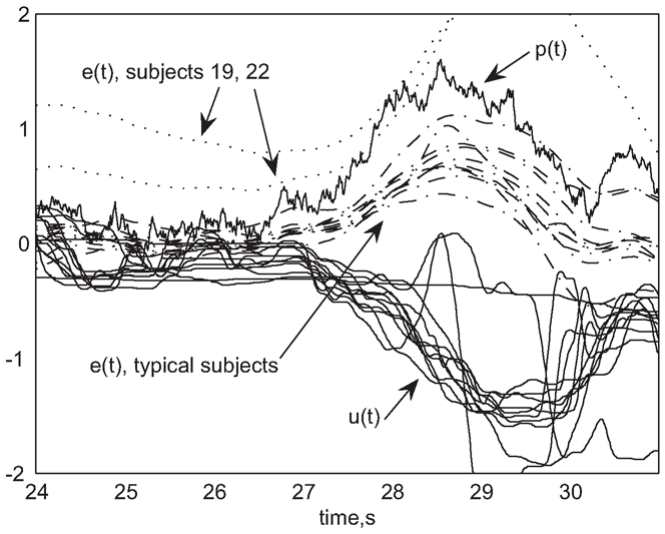
Time series data. Representative time series data: All elderly subjects, trial 7. Note identical 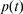 and two of the outlying elderly subjects, 19 and 22.

The experimental procedure was identical to that described in [25]. The paradigm is compensatory tracking with a controlled dynamical system implemented in software. A band limited Gaussian perturbation [36] continuously moves a displayed cursor on a horizontal line and the subject is asked to provide a corrective control input to make the cursor track the stationary midpoint of the line. Ideally the subject would provide an input that perfectly canceled the perturbation, but they cannot due to lack of foreknowledge of the perturbation, delays, and noise in the sensorimotor system. Fig. 2 shows a brief representative section of the time histories of the perturbation 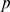, the control input 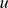, and the displayed cursor error 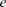.

The cursor's motion results from simple but marginally unstable dynamics:
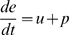
(1)


Equation 1 states that the horizontal velocity of the cursor 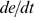 is the sum of the subject's control input 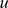 (defined as the left-right deviation of the manipulandum from its initial position) and the software-supplied perturbation 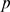. Thus the horizontal position of the cursor (i.e., error 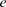) is the time integral of this velocity. These cursor dynamics are marginally unstable, similar to [37]. They resemble driving a car on a windy day such that, if the subject does not correct, then the cursor will wander off the screen in approximately 10 seconds. These dynamics are a compromise between the static 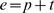 and the excessively difficult 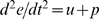, for which total loss of control was seen even in some young subjects.

The data for eleven of the young subjects appeared in [25]. Repeated testing was performed eight months later on eight of the young subjects as described in [25]. Subjects 1 and 10 participated and no longer displayed the outlying behavior described in this paper, and other subjects remained well described by the optimal control model. All the young subject data used in this paper for comparison to the elderly is from the first session.

### Introduction to the optimal control modeling approach

**Figure 3:**
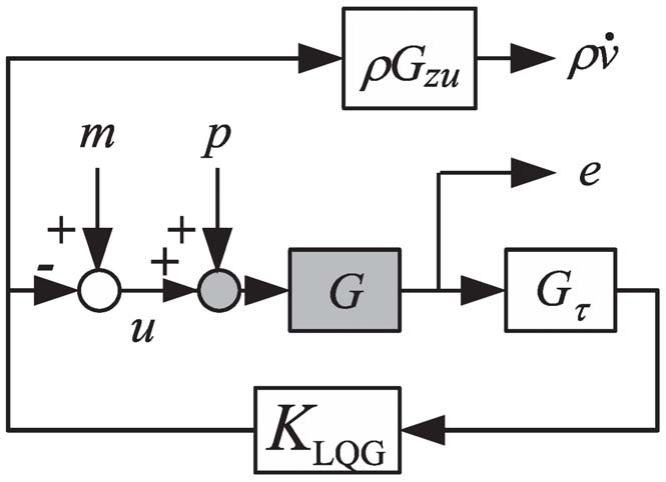
Model structure. This block diagram shows the closed loop system model for the optimal control approach. Only the shaded parts are physically outside of the subject: the controlled dynamics 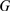 and the addition of the perturbation 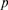. Endogenous noise is represented by 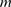. The displayed cursor error 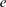 is delayed by 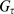. The delayed signal acts on the optimal controller 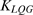 that outputs an ideal control input 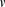. The expected sum of the squares of the outputs in the upper right is minimized by the choice of 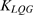. The weighting and differentiation in the cost function are implemented by 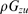. The latency parameter affects 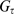, the control cost parameter appears directly, and the noise parameters affect the statistics of 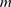.

We used a computational model to improve the precision of the response latency estimates and to investigate quantitatively the slowing of the post-onset response. The structure of this continuous linear feedback control task is suited to modeling using concepts from Linear Quadratic Gaussian (LQG) optimal control [38]. We need only make standard assumptions of quadratic optimality and additive Gaussian noise, and minimalist assumptions about a feedback loop structure as shown in Fig. 3, to arrive at our modeling framework. The quadratic optimality assumption is that the expected value of the following cost, integrated over an arbitrarily long time frame, is to be minimized:
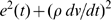
(2)where 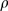 is a weight, 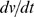 is the time derivative of the ideal control input (that is, hand motion 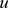 before adding endogenous noise 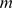), and 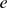 is the tracking error. The resulting LQG control strategy is to choose a control input by multiplying a problem-dependent static gain matrix and the Bayesian optimal estimate of the state of the system. These are referred to as the Linear Quadratic Regulator (LQR) gain matrix and the Kalman state estimate [38]. For a survey of applications of optimality as an organizing principle of animal behavior see [16],[17]. This approach is particularly relevant to the study of sensorimotor behavior because the delays and noises under which the task is performed are taken into account when computing the optimal control strategy. The use of this model to analyze visuomotor response is not a claim that it is the best possible model; its advantages are simplicity and parsimony [25]. We have not found a better model for response in our task in the literature, and we show later that a standard way of forming linear model fits (without any restrictions to optimality) does not do substantially better.

The standard LQG model introduced above is based on an additive noise model. It is well known that sensorimotor noise is signal-dependent, that is, its variance is a function of the amplitude of the corrupted signal [8],[11]. Considering the effects of signal-dependent noise will require us to make some mild assumptions, but ultimately leave us with the same model as the additive noise model, with a different interpretation of the 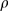 parameter. The remainder of this paragraph describes our approach. The most straightforward model of signal-dependent noise is to assume that the variance is proportional to the variance of the corrupted signal, that is, multiplicative noise. Two approaches may then be taken to solve the control design problem: either adopt time-varying control policies [18], or restrict ones' attention to much simpler time-invariant control strategies and examine the role of multiplicative noise in that tractable case. We do the latter. The well-known principle that estimation and control can be treated separately for the additive noise LQG problem does not hold for signal dependent noise [18], but it proves tractable and consistent with the data to continue treating the problem as having separate control and estimation components. First, in terms of optimal control given a state measurement, it has been shown that under the relatively mild time-invariance and multiplicative noise assumptions, control cost and multiplicative noise levels have a summed joint effect during optimal LQR control design [39]: a positive coefficient describing the intensity of the multiplicative noise is added to a freely chosen positive control cost during control design. This leads to a situation where we cannot disambiguate these two effects during model fitting unless we make a poor assumption that the noise is purely multiplicative. Second, in terms of estimation, the restriction to time-invariant control implies a time-invariant Kalman filter, which is equivalent to designing the filter with some appropriate level of additive noise. Ultimately, the approach described in this paper is to note that the tractable approximate approach to signal-dependent noise under mild assumptions is to use the same model as in the additive noise case, and then to note that the remaining difference is only in the interpretation of the parameter 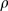: in an additive noise model, it is purely a freely chosen control cost, while in the approximate time-invariant solution to the multiplicative noise model, the parameter 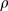 is the sum of a coefficient of noise intensity, and a freely chosen control cost.

The optimal control model structure is shown in Fig. 3 and its components are described in the caption. In our approach, the four parameters used to fit the optimal control model to the data from any given trial are:

1. response latency
2. intensity of the endogenous noise 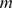 - this measures the signal variance per unit of bandwidth. The bandwidth is the next parameter.
3. endogenous noise bandwidth [38], 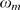 - the endogenous noise bandwidth is implemented by a prefilter on 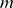 that is not shown in Fig. 3.
4. a parameter 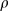 that includes the effects of “control cost” (a free design variable expressing aversion to motion) and multiplicative or “signal dependent” noise [8],[11], as described earlier

Once we fit parameters in the assumed modeling framework, the model is fully defined and we can predict properties of the control output 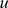. Iterative Nelder-Mead [40] minimization of the squared discrepancy between predicted and observed subject response was used to fit models. The resulting controllers have nine states because the plant has nine states (the delay is modeled with a fourth order approximation, one state in the computer, three states in noise filters, and one state in 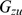), but less than five states are significant in the fitted controllers, based on analysis of Hankel Singular Values (HSVs) [38].

## Results

The first two results make no use of the optimal control model. The third result is a set of tests of the relevance of the optimal control model. The remaining results involve properties of the fitted optimal control models.

### No change in response latency

To determine the response latency in this task, we examined the cross correlation of the random signal driving the perturbation, and the subjects' response. The cross correlation [36] is a simple phenomenological way to analyze the temporal relationships between the discretized signals 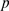 (the perturbation) and 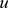 (the control input or “correction” by the subject) without making any modeling assumptions. The perturbation 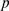 was a discrete-time approximation to band-limited Gaussian noise [36], created by passing a sequence of normally distributed random numbers (discrete time white noise) through a first order filter 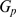 with a cutoff frequency of 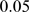 Hz. This filter is required to ensure that the perturbation is not “too jumpy” for the subject to react. This filter induces autocorrelation in 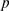 [36], thus in our analysis we use the pre-filtered or “whitened” [36] signal, 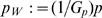 to remove spurious effects. We use indexing subscripts to denote samples at time 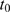 through 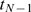 and compute the cross-correlation
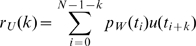
(3)Using the cross correlation to infer response latency is straightforward because it wanders randomly near zero for low values of lag index 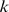 until the effects of the perturbation 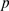 are seen in 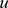. These effects occur because the subject attempts to negate the effects of 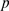. Therefore the cross-correlation begins to show a negative (i.e., corrective) trend at a value of 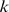 corresponding to the response latency, and we use this to measure latency.

**Figure 4:**
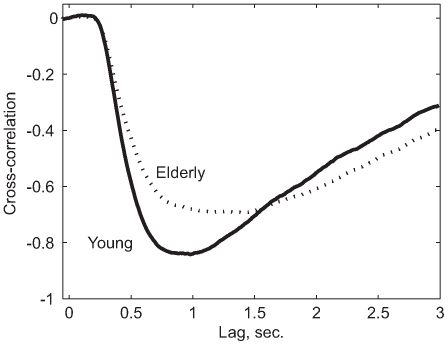
Cross-correlations. The mean cross-correlations for young and elderly show that there is no aggregate increase in response latency. This analysis is free of assumptions and makes it clear that there is a change in the post-onset response, but no change in the response latency. We removed spurious effects by subtracting 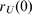 from each trial. The effects of perturbations on response persist longer when post onset response is slow, as seen here in elderly data. These averages were obtained at each lag value by averaging across trials 3–10 for all subjects, and then averaging across subjects.

Application of our model-free cross-correlation method yields mean inferred response latencies 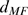 of 267 and 263 ms for young and elderly subjects. The precision of the inferred quantities is evidenced by the median intrasubject standard deviations of 36 and 44 ms. A two-tailed t-test [41] was used to compare the 12 young to the 12 elderly using the mean from each subject to avoid repeated measures bias. The measurements' distribution was consistent with normality with or without using logarithms. The t-test indicates that the 4 ms difference between the two means is not statistically significant 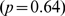. The averaged cross-correlation data of young and elderly subjects is presented in Fig. 4.

### The elderly perform worse: slower and with increased noise

**Figure 5:**
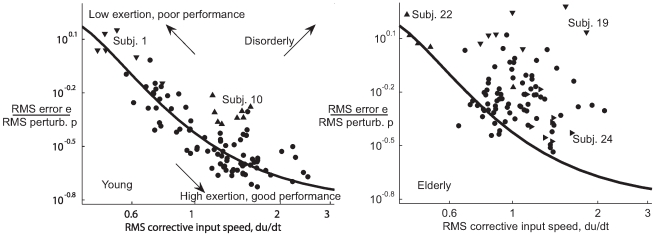
Effort/performance tradeoff. The left panel presents normalized RMS tracking error 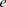 against RMS control input velocity 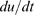 for young subjects (1–12), showing the existence of a tradeoff between effort and performance. The displayed line traces an effort/performance tradeoff implied by the model, and borne out in the data for the young subjects. Subjects well fit by the optimal control model (see the optimal control results section) are marked with circles. Subjects 1 and 10 (shown with triangles) are outliers in this panel and were not well fit by the optimal control model. They did not reproduce the outlying behavior shown here during repeated trials as shown in [25]. The effort/performance tradeoff line is obtained by varying the control cost parameter in the optimization while also adjusting the noise intensity to account for the multiplicative or “signal dependent” nature of the noise. The right panel shows that the elderly (13–24) are generally unable to obtain as favorable an effort-performance tradeoff. The displayed reference line is not a fit to the data. Rather, it is the same line as in the young data, provided for comparison. Again subjects well fit by an optimal control model are indicated with circles, while subjects 19, 22, and 24 are indicated with triangles.

We observed increased disorder in the response of elderly subjects. Good performance in these tests corresponds to low root mean squared (RMS) cursor error 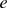. A subject's behavior is more efficient if they obtain the same level of performance using less corrective motion, that is, low RMS control input velocity 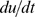. Significantly, young subjects choose different strategies in this task, leading to a Pareto optimal curve in Fig. 5. We calculated the line fit to the data of the young subjects using the optimal control model by varying the control cost and noise intensity parameters in a coupled way according to the multiplicative/signal-dependent nature of noise in sensorimotor tasks [9],[11]. Despite uniform physiological health among the young, and the instructions to “keep the error as small as possible,” some provided less corrective effort and tolerated larger amounts of error. In repeated trials spaced months apart, some subjects shifted location along this Pareto front, and the outlying young subjects 1 and 10 moved onto this front. In Fig. 5 the elderly fall almost completely to one side of the curve fit to the young. When compared to the young, they move their hand more but accomplish less. Without assuming any model, it is reasonable to conclude that this is not intentional, and that it demonstrates increased noise or disorder in their response.

The elderly also have a lower average RMS control input rate 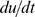 - that is, they move more slowly. The effect is visible in Fig. 5. The 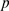 value of a two-tailed t-test on the logarithm of the subject means across twelve young vs. twelve old subjects is 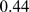. Removing subjects that were always poorly fit by the optimal control model (1,10,19,24) yields 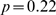. The existence of age related slowing is well established elsewhere. In this experiment, it is reasonable to suppose that the high 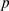 value results from the effort/performance tradeoff seen in the young subjects' data in Fig. 5.

### The optimal control model is relevant to all young and most elderly subjects' behavior

Despite the use of a fitting process, there exist opportunities for the optimal control model to be tested. To be precise, the model parametrizes an infinitesimal fraction of all possible functional control strategies, and thus despite the fitting process it has opportunities to be inconsistent with either the data or with the results of accepted analysis methods. These are enumerated below.

**Figure 6:**
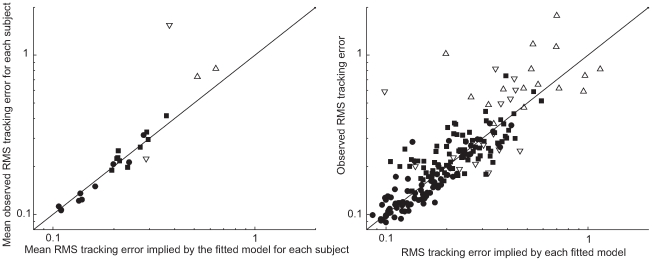
Observed vs. implied tracking error. The observed RMS tracking errors are consistent with those implied by the fitted model. Agreement is excellent if results are averaged across trials for each subject, as shown on the left. The amount of experimental scatter can be seen on the right. One subject-trial is off the right hand panel. Young subjects are denoted with circles unless they were poorly fit by the optimal control model (1,10), then they are shown with downward pointing triangles. Elderly subjects are denoted by squares except the poorly fit 19 and 24 denoted by upright triangles.

First, a fitted model implies a certain level of RMS cursor error 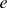. This is not a prediction outside of the data set, as all the measured variables are coupled by the feedback loop. However, it is evident from theory and experiment that it is possible for the observed RMS 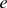 to be significantly different from that which would be expected based on the fitted model, and this provides an opportunity to prove the model false. This is can be understood with the following equations, based on zero assumed initial conditions:
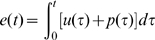
(4)

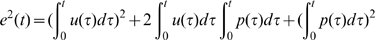
(5)This indicates that the accuracy of the model's predicted RMS error level 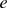 rests on the accuracy of its model of the interaction of 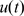 and 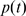 - that is, the subject response to the perturbation. By restricting our models to be optimal controllers, we are restricting the choices available to our fitting method to represent this interaction. The ratio of predicted and observed RMS 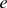 is near one as shown in Fig. 6. There is some loss of accuracy due to the use of a statistical model of endogenous noise over a finite time frame. Again, the outlying behavior by young subjects 1 and 10 was not reproduced on repeated testing.

Second, the model would be inconsistent with the data if the fitted models did not obtain significantly different parameters for different subjects (consistent with their supposed meaning), in cases where direct inspection of the data shows that such differences exist. This is not a difficult test, as the modeling method is of course designed to capture this difference, but it is still useful to inspect the results. Summary statistics of the model parameters in Table 1 indicate that the method is able to detect intersubject differences and yield sensible results. The base 10 logarithm of all parameters was taken for normality. The table gives mean values for the young and old, the aggregate SD, the median intra-subject SD, and the median intra-trial SD. After the four parameters, a parameter proportional to motor noise amplitude normalized to control input amplitude is given. This demonstrates that the age-related reduction in signal to noise ratio implied by Fig. 5 is observed in our parameters. The two-tailed t-test p-value for logarithms of the twelve young subject means vs. twelve elderly subject means for this quantity is 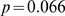. It decreases to 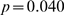 if the subjects that were poorly fit by the optimal control model (1,10,19,24) are excluded.

**Table 1:** Inferred parameter summary.

parameter	mean young	mean elderly	SD	MISSD	MITSD
control cost/mult. noise 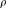	−0.7257	−0.3010	0.4552	0.1817	0.4446
latency	−0.5879	−0.6252	0.1109	0.0268	0.1099
noise int.	−2.7556	−2.6408	0.5756	0.4205	0.5729
noise bandwidth (BW)	0.9495	0.9039	0.2713	0.2428	0.2668
BW*int./(Mean Sq. 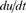 )	−1.8566	−1.7332	0.3745	0.2607	0.3448

Statistics of inferred parameters and a derived measure of multiplicative noise intensity. Base-10 logarithms are used for normality. MISSD is median intra-subject SD, and MITSD is median intra-trial SD across trials 3–10. BW is bandwidth in radians per second of the endogenous noise filter 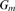, and int. is the intensity (variance per bandwidth) of the endogenous noise before that filter. The quantity (BW*int./(Mean Sq. 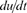)) is the power of the endogenous noise relative to the power of the exciting signal based on the fitted parameters.

**Figure 7:**
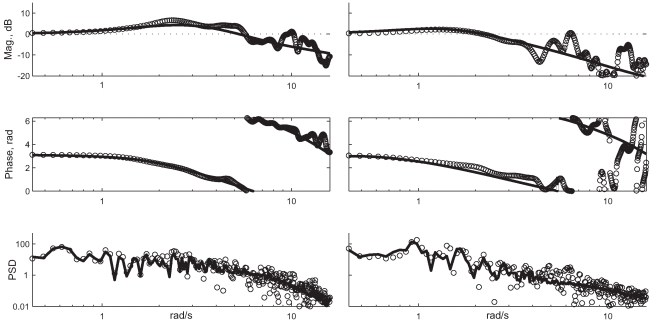
Typical spectral fit. This shows representative spectral fits for young (left) and elderly (right) subjects. Discrete data points are circles, and lines are fits. They were selected by choosing the trials with median fit cost from each group. The top panel is the magnitude of the closed loop transfer function 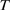 compared to the experimental cross spectrum of 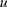 and 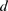. The next panel is the phase of the same quantity. It decreases, wrapping around, as the effects of latency and limited bandwidth build up. The bottom panel is the fitted and observed power spectral density of the control input 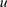. The apparent overfitting is due to the model fitting process' knowledge of the perturbation, and the dominance of the low frequency behavior by response to the known perturbation rather than endogenous noise.

Third, the agreement between observed and fitted Fourier transformed auto- and cross- correlations [36] of input and perturbation for the optimal control model are good. It is difficult to make this statement precise because spectral analysis is an engineering technique to guide model creation rather than a statistical tool for testing hypotheses [36]. There exists a metric resembling the 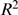 measure, coherence, but it is valid only for a general form of empirical linear model fitting [36]. In Fig. 7 we show spectral data from the trials with median fit cost from young and elderly subjects. The spectral fits of (1,10,19,24) were generally poor and those of 22 were poor for some trials. *Remarks:* There exist multi-taper methods for constructing experimental spectral estimates with confidence intervals [42] but these rely heavily on assumptions about stationarity of statistical processes. Due to the signal-dependence of noise, these assumptions are invalid. Another commonly used measure of linear dynamical model fit quality is coherence. Conceptually, this is a frequency-dependent 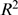-type goodness-of-linear-fit measure [42] but coherence is well-defined (such that it falls in [0,1]) only for empirical data smoothed using certain tapers [36], and not for arbitrary linear models including ours. The experimental spectral data shown in this paper are such smoothed empirical data, and the coherence is invariably nearly one at low frequency and nearly zero over 10 radians per second. In terms of our model, this loss of coherence is due to dominance of endogenous noise. Were the behavior at higher frequencies actually productively structured, and the label of endogenous noise therefore incorrect, then we would expect to see over-predicted RMS error levels. This is not the case generally. Thus low coherence at high frequencies is not a failing of our model, but a manifestation of the limited ability of the subjects to execute productive motion at those frequencies.

**Figure 8:**
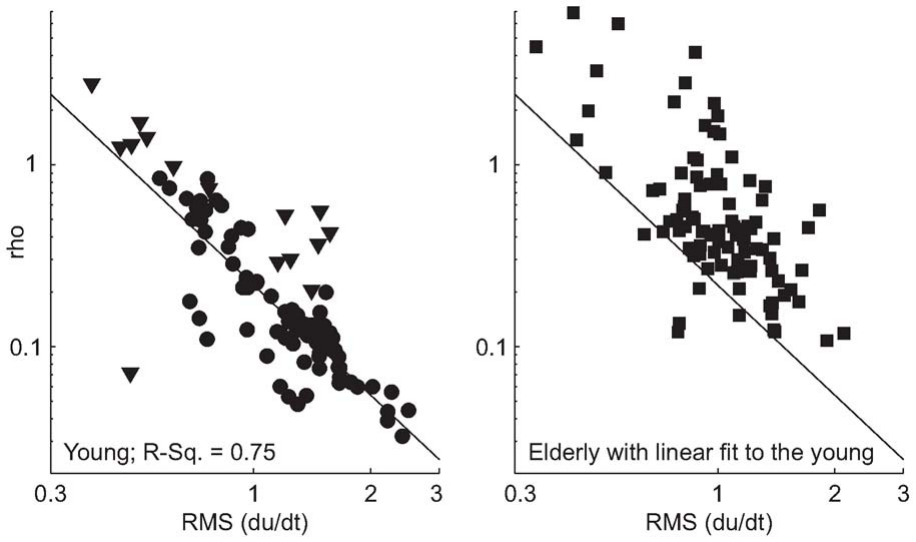
Relationship of 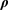 to input velocity. The left panel shows that among a uniformly healthy young population whose multiplicative noise characteristics can be expected to be uniform, a relationship between the control cost/multiplicative noise parameter 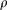 and observed RMS 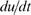 exists as expected. Data points from outlying subjects 1 and 10 are shown with triangles and omitted from the displayed empirical least-squares linear fit. The increase in this parameter at any given level of RMS 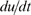 for the elderly is shown in the right frame. This is consistent with increased noise.

Fourth, the optimal control model would be in doubt if its inferred latency values were inconsistent with the result from the cross-correlation method; this is not the case, as described in detail in the next subsection. Along similar lines, the increase in disorder visible in Fig. 5 should be captured in our model's parameters as increased multiplicative noise and therefore reflected in the fitted 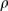 parameter. Specifically, direct inspection of the data in the left panel of Fig. 5 shows significant differences in RMS control input 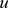 that should be correlated with age-related changes in the multiplicative noise/control cost parameter 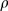. Fig. 8 shows that the expected relationship holds for the young, where it is reasonable to assume that the effect of multiplicative noise on this parameter is relatively uniform due to uniform health, and variations in 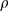 result only from altered willingness to expend effort. What is more interesting is that Fig. 7 also shows that 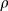 tends to be larger for the elderly given some level of observed RMS control input 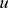. This is consistent with the empirical observation of increased multiplicative noise.

Fifth, the optimal control model would be in doubt if more general linear system identification techniques were able to better fit the data. Even with a restriction to the set of all stabilizing linear controllers with similar state dimension, the optimal controllers represent an infinitesimal fraction of that set, due to restrictions on the LQG cost function implied by our parametrization [38]. We therefore compared the fit costs attained by our model fitting method to those obtained using the more general n4sid [43] model fitting method available in Matlab [40]. The mean ratio of the fit costs was 1.009, and the standard deviation was 0.0062.

Sixth, the optimal control model predicts that there is a volitional degree of freedom in response, parametrized by the control cost component of the parameter 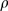. Therefore a population with uniform multiplicative noise properties should fall along a corresponding Pareto front. This is indeed observed among the young as shown in Fig. 5.

Last, we have a catch-all that if some phenomenon exists in the data that is not qualitatively consistent with the model, the model is flawed in that sense. We observe intermittent periods of stillness 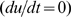 that are not predicted by our model, as is visible in Fig. 2. These tended to occur in lower-performing subjects, and became very pronounced for the worst-performing elderly subjects. They appear to be either periods of inattention or control deadbands or deadzones similar to those reported in [44]. This discrepancy reflects the approximate nature of our assumption of quadratic optimality - evidently, if the appropriate corrective motion is sufficiently small, it is sometimes ignored. This flaw does not seem important to the phenomena we address in this paper.

Besides consistency with the data, a good model should be parsimonious, and we demonstrate this for young subjects in [25]. Delayed proportional control of limited bandwidth would be a conceptually simpler model of response. For high values of the multiplicative noise/control cost parameter 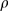, our control model becomes very similar to this strategy, and indeed the data for lower performing subjects is well described in this way. Proposed methods to fit both optimal and less parsimonious non-optimal models to the behavior of healthy young subjects are surveyed in [45]. Rather than adopting different modeling approaches for different cohorts we simply use the optimal approach throughout.

Based on the above results, we conclude that the optimal control model is appropriate as a model of the behavior of all young subjects, and for the elderly except subjects 19, 24, and marginally 22. The model was consistent with the behavior of all but two young subjects during a first session, and with the behavior of all young subjects upon repeated testing [25].

### Latency values are more precise with the optimal control model

**Figure 9:**
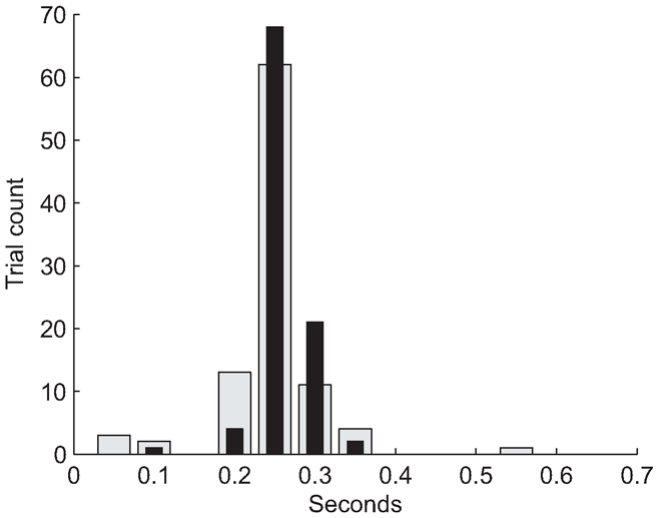
Latency from the optimal control model. Inferred latencies using the optimal control model, confirming the absence of increased response latency in the elderly. The young are thin black lines, the elderly are wide gray bars. This is a histogram, meaning that we compared inferred latencies to a set of evenly spaced reference values, and incremented a count of subject trials corresponding to the nearest reference value.

The inferred mean latencies using the model-based method for young and elderly groups are 260 ms and 247 ms, therefore we cannot attribute elderly impairment to increased response latency. The median intra-subject standard deviations are 14 ms for the young and 20 ms for the elderly, which is significantly less that the variability of the cross-correlation method applied to the same data. The difference in means is not significant: Averaging inferred latencies within each subject, grouping them into 12 young and 12 elderly, and applying the two-tailed t-test yields a p-value of 0.175. A histogram of the model-based inferred response latencies is presented in Fig. 9, aiding interpretation and confirming normality.

### The optimal control model predicts slowing as a result of increased noise

This is a computational result obtained by taking the models fitted to the young and increasing the noise bandwidth parameter, the noise intensity parameter, or the multiplicative noise/control cost parameter 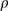. The magnitude of the predicted closed loop subject response to the perturbation decreases in each case, and particularly at higher frequencies. This implies slowed response. Within the structure of the optimal controller, this is associated with smaller elements in the LQR gain matrix (in the case of 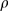) and longer timescales in the Kalman Filter (in the case of the noise parameters). This is consistent with both classic results concerning signal-dependent or multiplicative noise [7],[8],[11],[39] and the intuitive suggestion about averaging timescales in [2].

Within our data, the behavioral changes seen in nine of the twelve elderly can be replicated by retuning of the optimal models found in the young to increase noise. This follows directly from the fact that nine of twelve elderly subjects' behavior is well fit by our model without significant changes from latency values from the young group.

### Optimal models fitted to the elderly are less complex

**Figure 10:**
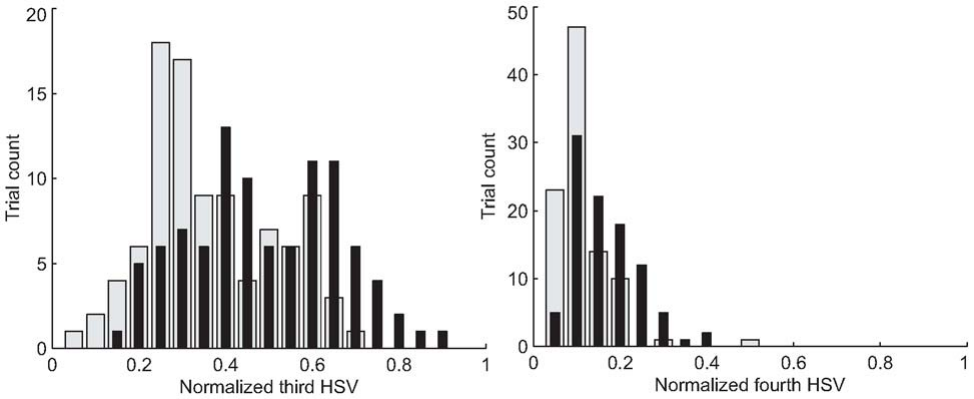
Complexity: Hankel Singular Values. These histograms of normalized third and fourth Hankel Singular Values (HSVs) of inferred controllers suggest that the complexity of the behavior of the elderly is reduced (see the Discussion). The young are thin black lines, the elderly are wide gray bars. Fifth and higher HSVs are negligible.

We examined the normalized Hankel Singular Values (HSVs) of the optimal control models that we fit to the behavior of our subjects. We found that models fit to the elderly subjects have smaller third and fourth normalized HSVs as shown in histograms in Fig. 10. Taking the logarithms of the mean normalized third and fourth HSVs for each subject and again applying t-tests on young and elderly groups yields p-values of 0.018 and 0.026, indicating that simpler models are fitted by our method to the behavior of the elderly group. Higher HSVs were negligible.

## Discussion

The principal experimental result of this work is that age-related impairment in a dynamic compensatory tracking task takes the form of reduced efficiency of corrective motion without an increase in response latency. This is a robust result in that it can be obtained with a phenomenological cross-correlation method free of assumptions, and reproduced with greater precision by a more sophisticated model-based approach. The optimal control model presented in this paper has testable implications for behavior in this task as surveyed in the results. These indicate that it is relevant as a model of behavior for all young subjects and most elderly subjects. Therefore the model is relevant to analysis of at least the initial phases of age-related changes in sensorimotor response. We then have the computational result that if one adjusts the parameters used in the optimal control models fitted to the young to account for increased noise levels, the control strategy changes in a way consistent with slowing. Thus analysis of the computational model is consistent with the hypothesis that slowing of post-onset response is an optimal adaptation to increased endogenous noise. This hypothesis has been suggested before without formulating an explicit model [2]. This hypothesis has also been suggested based on an analysis involving hypothesized submovements and empirical speed-accuracy tradeoffs [7], rather than Bayesian and quadratic optimality.

Both our empirical and model-based results are inconsistent with the generalized slowing hypothesis of aging as reviewed in [1],[28]. Fig. 5 shows that the elderly are unable to obtain as favorable a speed/accuracy tradeoff as the young. This is inconsistent with the hypothesis that slowing might be due to a choice involving this tradeoff put forward in [46].

Elderly impairment is shown in Fig. 5 to manifest as inefficient, disorderly movement; therefore we try to clarify the definition and role of endogenous noise in this paper. As described in the introduction, we define “endogenous noise” as deviations from optimal behavior, and therefore include suboptimal performance due to sensory, motor, conduction, or neural processing imperfections. A similar definition is unavoidable when defining noise in dynamic response, and it is best viewed as simply self-consistent. For example, in static force production tasks or target reaching tasks, there is implicitly a reasonable and simple model of what the subject “wants” to do, and noise is defined similarly as a deviation of behavior from that model. Any distinction between (a) involuntary cortical “dysfunction,” and (b) voluntary suboptimal or non-functional behavior, is bound to be scientifically unsatisfactory absent some quantitative measure of subject intent. Therefore we make no such distinction. This does not mean that our claim that the elderly have worsened endogenous signal-to-noise rests on our model. Instead we refer the reader to the deviation of the elderly from the Pareto front in Fig. 5, an indication that the effort/performance tradeoff has worsened with age. This can only be due to a worsened mixture of productive behavior (i.e. signal) to unproductive behavior (noise).

We emphasize that optimal modeling does not assume that the subjects behave perfectly. It is better understood as a modeling approach that treats behavior as the sum of the effects of an optimal controller and a random noise source, acting in a feedback loop. The series of successful predictions of the optimal model were detailed in the Results section. Importantly, our report of outlying subjects shows that significantly suboptimal strategies are viable but atypical, and thus that our assumed form of optimality is not a trivial implication of success in the task.

Adaptation of motor control strategies with age has been reported elsewhere [32]. Evidence for altered control strategies in the elderly is presented in [47],which showed that different neural adjustments are used by the elderly during learning in an isometric contraction reference tracking task. In addition, [48] reports the use of co-contraction to suppress noise using EMG measurements. This co-contraction was associated with slowing, and thus suggests one way to implement a slowed and less complex controller.

We investigated the possibility that reduced complexity of elderly control strategies in this task caused reductions in response latency that offset expected aging effects, as suggested by well-documented age-complexity-latency interactions [1],[4],[5],[27],[28],[49],[50]. Complexity has proven to be a “slippery” concept in the psychology of aging [49]. Furthermore, quantifying complexity in a dynamic task is intrinsically more difficult that in a discrete choice task. In our experimental paradigm, unlike in a choice reaction task, the complexity of the subject's response is not constrained. We consider our task as at least as complex as a one-bit choice reaction task in the sense that there are two possible directions that the subject may move their hand, and furthermore the amplitude of the motion must be determined. There have been reports of methods to measure the complexity in a time series (the data format of our dynamic perturbation-rejection task) such as entropy based methods [50],[51]. In the time series entropy paradigm, complexity is a property of the signal rather than the neural control system, and it is determined by the output signal of the system of interest. These entropy methods based on measuring only the output of a system imply a paradoxical assertion that a system that outputs noise (such as thermal noise from a resistor) is more complex than a system that outputs structured signals (such as a brain). The paradox can be resolved by viewing complexity as a property of the system rather than of the signal. In the experiment, we can only infer a system model from the signals input to and output by the subject. This justifies treating complexity in terms of systems' input-output relationships as identified in dynamical modeling. The linearity of optimal response in our experimental paradigm made available the well-developed tools of linear system theory, in particular, the Hankel Singular Value (HSV). Our results show that the elderly employ strategies of reduced controller complexity according to this metric. We report a computational result but draw no immediate physiological conclusions about this for two reasons. First, we can only speculate on the relevance of this result to response latency because the dependent variables of response latency and inferred HSVs have too much variance (even within the homogenous young group) to allow us to demonstrate a HSV-latency effect within our data. Second, higher order terms in computational models can display epiphenomena and generally call for independent confirmation.

Concepts from our modeling approach resemble those in contemporary work involving Parkinson's disease. It was found in [52] that in a pointing task with a penalty associated with deviations from a desired time-to-completion, affected patients were slower than healthy subjects to adjust to requests for increased speed - without any change in endpoint accuracy. This result contrasts with our empirical observation of increased disorder in elderly subjects, though the comparison is necessarily indirect due to the differences in tasks and measures.

In conclusion, the major contribution of the optimal control model is that it quantitatively specifies the extent to which subjects should use slower control strategies to reduce the effects of noise. This is subtly different from less mathematically precise ways of arriving at this reasonable strategy. Specifically our model holds that the slowing should take the form of longer timescales during the response dynamics, and makes no prediction of longer latency until the onset of these response dynamics. This is consistent with the surprising experimental result that there is no increase in latency until onset of response in this relatively complex task.

## Methods

### Ethics statement

All subjects completed consent forms approved by Cornell University's Committee on Human Subjects and brief health questionnaires.

### Experimental method

The young subjects numbered 1 through 12 were healthy volunteers between 20 and 32 years of age. The elderly subjects numbered 13 through 24 were between 65 and 92 years of age. The manipulandum was a calibrated optical mouse modified to only record lateral hand motion, and mounted on ball bearings to reduce friction. Subjects were free to use the hand with which they felt most comfortable. Two self-reported left-handed subjects chose to use their right hands because they are accustomed to using computer mice with the right hand. This did not seem to affect the data, as the outliers were all using their right-dominant hand. There were ten 60-second trials. The first 9 seconds of data from each trial were discarded. Trials started every two minutes to prevent fatigue. Each trial consisted of a different random perturbation time history, and all subjects had the same perturbation time histories in the same order. The results of the first two trials are neglected to allow for learning effects. All young and all but one elderly subjects' behavior converged in this time, as measured by RMS cursor error and input velocity. The software sampled the control input 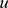, added the perturbation 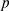, and updated error 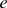 at a rate of 100 samples/second. This rate ensures that closed loop behavior is unaffected by software-induced delay, and that measurement resolution covers the timescales of motor behavior in the hand and arm.

### Scalar latency measurements from cross-correlation data

To assign a specific time value for response latency based on cross correlations, we looked at the peak of the second derivative of the cross correlation with respect to lag index 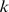 (see Eqn. 3. Because derivatives introduce spurious numerical noise, we smoothed 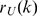 by forward and backwards discrete filtering to obtain 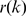 before calculating the second derivative. We used a second order Butterworth filter [40] with a 20 Hz cutoff frequency.

### Modeling approach

Consistent with today's literature, we assume quadratic optimality and Gaussian noises. We present a linear plant to be controlled. The dynamics of our task and the basic dynamics assumed to exist in the human are jointly described by the state space matrix equation:
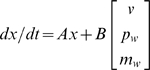
(6)

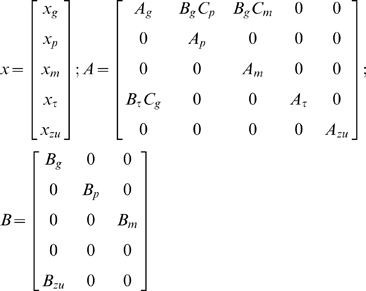
(7)

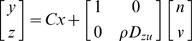
(8)

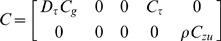
(9)The state 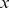 includes states from each sub-system: a state 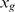 from the controlled dynamics in the software, one state 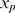 for the pre-filter on the external perturbation, two states (vector 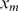) for the pre-filter on the motor noise, four states (vector 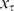) for a fourth order Pade approximation to some delay that is assumed to exist in the human, and one state 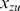 needed to implement differentiation of the control input 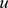 over the frequency range of interest for use in the cost function. The inputs to (7) are an ideal noise-free control input 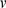, a pre-filter white perturbation 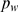 with known statistical properties, and a pre-filter white endogenous noise 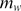 with known intensity. Intensity is defined as variance per sampled bandwidth. The measurement output 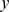 available to the subject is 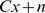, where 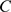 is such that the states involved in the approximation to the delay affect the measurement so that the cursor error 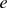 (identical to 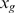 in the general notation) can only be observed after the delay. The measurement noise is 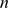. Each of the subsystems is represented above in standard state-space form by 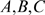, and 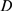 matrices [38] with appropriate subscripting. The 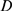 matrices are zero except for 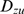 and 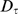. For example the perturbation pre-filter subsystem in isolation is given by
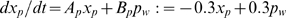
(10)

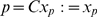
(11)The above equations are in matrix form; sometimes the matrices are scalars, and sometimes 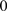 is actually a matrix of zeros, as appropriate. The cursor dynamics are given by 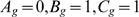 so that 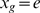. The control-effort differentiation dynamics are given by 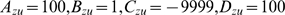. These dynamics implement differentiation of the control input across the frequency range of interest while avoiding technical problems during LQG synthesis.

The other sub-systems (endogenous noise prefiltering 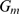 and latency 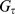) are now presented. The pre-filters 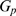 and 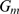 allow us to solve the relevant equations without unreasonably assuming white noise disturbances in the physical system. The endogenous noise prefilter 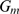 is a critically damped second order system
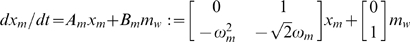
(12)

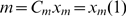
(13)Our approach lumps all latencies in the system into one system 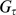, but in our simple single-input-single-output task this makes no difference. The state space matrices 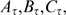 and 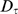 for 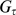 vary depending on the parameter, and are non-unique and unenlightening. This is tolerable because they are easily created with commercial software implementation of the Pade approximation [40] for any given latency value and approximation order.

The measurement noise 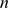 was estimated to be at a negligible level for all subjects by having subjects read the instructions in a very small font. If the measurement noise is taken to be white, this acuity corresponded to a level of noise low enough that optimal response is insensitive to it. Non-white measurement noise models were not pursued because their effects are ultimately indistinguishable in their effects on subject response 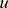 from changes in the main endogenous noise 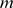.

The matrix 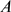 is large and complex, but commercial software can assemble it automatically given a block diagram and straightforward equations for its components [40].

We assume that the subject behaves so as to minimize the following quadratic cost:
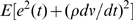
(14)where 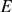 is the expectation operator [38]. This assumption is chosen because it is tractable, consistent with contemporary methods, and a simple way to capture the concept of “reasonable” control strategies. In conjunction with the linear model, this assumption implies Linear Quadratic Gaussian (LQG) optimal control [38]. LQG control is implemented by the series combination of a Kalman filter (a Bayesian optimal state estimator) and a controller that minimizes a cost defined as a quadratic function of input and state variables. The Kalman filter is of the form
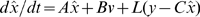
(15)This equation holds that the state estimate 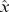 evolves based on a known state space dynamical matrix 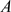, the input matrix 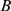 describing the effects of control inputs on the state, and a Kalman gain 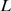 times the difference between the observed measurement 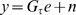 and the expected measurement. The assumption that the 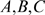 matrices and the noise statistics are known implies an assumption that response is practiced and an internal model is formed. The Kalman gain 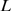 is obtained by solving a Riccati equation involving 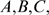 and noise variances [38], a process automated in commercial software [40]. The ideal control input 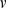 is then
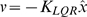
(16)where 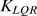 is a static gain matrix, similarly obtained by solving a Riccati equation [38] involving 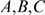 and the control cost.

Our model of the response, 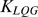, is then defined by
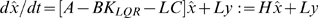
(17)

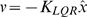
(18)

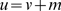
(19)


Forms of LQG control are widely used in both control engineering and the modeling of sensorimotor response, as described in [9],[17],[22],[25]. In any such approach, the optimal controller is uniquely defined when the controlled system and the statistical properties of all disturbances are specified.

Our model fitting procedure is done in the frequency domain. It is technically equivalent to using a weighted function of the discrepancy between the experimental and predicted values of the cross- correlation of 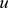 and 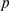 as well as those of the autocorrelation of 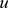 in the correlation- or lag-domain. We chose to use the frequency domain for easy comparison of the smoothed experimental cross correlation to the predicted closed loop transfer function 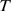 from 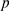 to 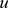, because the effects of added noise are visible in a straightforward way in the power spectrum of 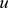, and because it lends itself to frequency weighting in the cost function as explained later. A purely time domain fitting process was not used because the expected effect of endogenous noise on the time series is zero, making this key aspect of response invisible to the fitting process. The predicted closed loop transfer function 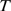 and predicted power spectrum 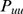 are specified by our model and the four parameters. In order to compare it to experimental data they can be evaluated at the sampling rate 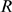 multiplied by the discrete frequencies 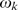 corresponding to the Discrete Fourier Transformed experimental data [36]. This is done in the weighted cost function
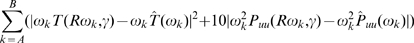
(20)In this cost function, 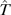 is the smoothed Discrete Fourier Transformed cross-correlation of 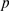 and 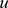, 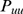 is the predicted power spectrum of 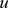, and 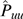 is the un-smoothed experimental power spectrum of 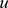 [36]. The parameter set is represented by 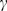. It is appropriate to not smooth the experimental power spectrum because response at low and middle frequencies is dominated by linear response to a perturbation that is known during analysis. The frequency weighting in the cost function emphasizes data at crucial frequencies near the closed loop bandwidth, rather than low frequency behavior that is not sensitive to key features such as latency, thereby reducing the variance of inferred parameters and the quality of the spectral fit in that frequency range. Results were insensitive to varying the weight of 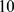 across the range of 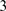 to 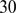; some weight is required to avoid over-fitting 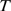 at the expense of 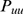.

The sensitivity of the predicted closed loop transfer function 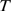 and the predicted power spectrum 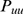 to the parameters can be summarized as follows. One can distinguish between direct effects that occur regardless of whether the control strategy adapts, and indirect effects due to changes in the control strategy that are assumed to result from the subjects' understanding of their own latency and endogenous noise. Our method assumes that both direct and indirect effects manifest in the data. Increasing the control cost/multiplicative noise parameter 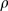 indirectly but strongly decreases 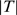 and 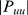 at all frequencies, especially high frequencies. This occurs solely through the its effect on 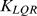. Increasing the latency parameter has a direct effect on the phase of 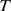 at higher frequencies, and indirect effects through the Kalman filter design that tend to cancel out some of the changes in phase of 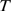 across a lower frequency range. These indirect effects are due to the effects of changing 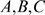 (which expresses changes in 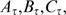 and 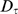) when solving the Riccati equations for the Kalman filter. Increasing the endogenous noise intensity parameter has a direct effect of increasing 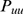, especially in a range near 1–3 radians per second depending on 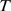, and an indirect effect of decreasing 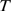 at higher frequencies, again through effects the Kalman filter design. Increasing the endogenous noise bandwidth parameter directly causes an increase in the predicted 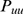 at higher frequencies and has relatively small indirect effects on 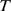, again through similar effects on the Kalman filter design.

Further details can be found in [25].

### The Hankel Singular Value

Our measure of control strategy complexity is size of the Hankel Singular Values (HSVs) [38] of the fitted optimal subject response. The number of significant HSVs is the effective state dimension or order of a linear system model. For example, one could take reasonably noisy data from a second order system and fit a higher order model to it, but the first two HSVs would be much larger than the rest. In this sense the method resembles Principal Components Analysis for linear dynamical systems. Where Principal Components Analysis involves the Singular Value Decomposition (SVD) of a covariance matrix, HSV analysis looks at the SVD of the product of the controllability and observability Gramians [38]. The controllability Gramian indicates the sensitivity of the system's states to the inputs, and the observability Gramian indicates the extent to which changing the system's states leads to measurable outputs. System states are variables that capture all information from the past relevant to future system behavior. State dimension is essentially identical to a concept already introduced to the aging literature as a measure of complexity, i.e., the number of dynamical degrees of freedom that can be regulated independently [49]. A system state variable is precisely such a dynamical degree of freedom.

The controllability Gramian of the LQG controller in Eqns. 17, 18 is
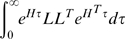
(21)The observability Gramian is
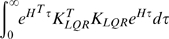
(22)


Without altering input-output relationships from 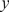 to 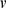, one can express the LQG controller using a linearly transformed state 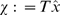 such that the state equations of 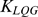 are expressed in so-called “balanced” form, characterized by the Gramians being equal, diagonal, and monotonically decreasing along the diagonal. This process is described in [38] and automated in commercial software [40]. Their SVD is then trivial, and the singular value corresponding to each state in 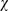 indicates its relevance to input-output behavior.

While, to our knowledge, we are the first to use HSVs in the neurophysiological field, they have a long history of use in system and control theory, where they are the standard method to make decisions on what order of model is required to approximate the behavior of dynamical systems [53]. It is often the case that a few states are influential and the remainder may be neglected.

Based on the preceding development, the complexity of implementing the inferred controllers may be quantified by examining the extent to which high order models are required to describe the behavior of the system - that is, whether the higher order HSVs are large. We normalized the magnitude of all HSVs by the subject's mean first HSV in order to eliminate the gross effect of altered gain, which we do not consider a form of complexity (omitting this refinement only amplifies the age differences). The dynamical significance of larger third and fourth HSVs in models fit to young subjects is that simpler low order models are less able to describe their response.

The HSVs are sensitive to all of our model parameters, but respond most strongly to multiplicative noise/control cost 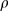: reductions in this parameter are associated with high order models. The HSVs are unique for a given set of the parameter values, because the parameter values fully define the modeled system, and the HSVs are a property of the system.
